# First case of *Propionibacterium acnes* urinary tract infection in a dog

**DOI:** 10.1186/s12917-015-0620-5

**Published:** 2015-12-21

**Authors:** Kazuki Harada, Takae Shimizu, Takeshi Tsuka, Tomohiro Imagawa, Takashi Takeuchi

**Affiliations:** Joint Department of Veterinary Medicine, Tottori University, Minami 4-101, Koyama-Cho, Tottori 680-8553 Japan

**Keywords:** *Propionibacterium acnes*, Dog, Urinary tract infection, Cystitis

## Abstract

**Background:**

*Propionibacterium acnes* has been rarely isolated as a commensal from dogs, but there is little evidence of pathogenicity. Urinary tract infections are common in dogs and are typically caused by various commensal bacteria. Here we present the first case report of a urinary tract infection caused by *P. acnes*.

**Case presentation:**

A 6-year-old female Japanese Shiba Inu was hospitalized for polyuria, polydipsia, and severe hematuria. At admission, blood tests revealed leukocytosis, slight anemia, decreased albumin, and slightly elevated blood urea nitrogen. Computerized tomography showed gas accumulation on the inner side of the bladder wall. Urinalysis revealed proteinuria and bilirubinuria without glycosuria. The urine sediment contained large numbers of erythrocytes and leukocytes. Additionally, rod-shaped bacteria were detected by Diff-Quik staining. Enrofloxacin and metronidazole were administered empirically; however, the renal function declined sharply and the patient died 2 days later. Bacteriological examination revealed that the causative agent was *Propionibacterium acnes*, which was identified as sequence type 53 via multilocus sequence typing. This isolate showed high susceptibility to ampicillin, amoxicillin/clavulanic acid, cefoxitin, imipenem, clindamycin, tetracycline, chloramphenicol, and enrofloxacin, but was resistant to metronidazole.

**Conclusion:**

To the best of our knowledge, this is the first case report of a dog with urinary tract infection caused by *P. acnes*.

## Background

*Propionibacterium acnes* is an aerotolerant, anaerobic, Gram-positive, rod-shaped bacterium commonly isolated from humans. It is often implicated in acne vulgaris and occasionally in postoperative and implant-associated infections [1]. In dogs, this bacterium can be isolated as a commensal from the skin [2], intestinal tract [3], and oral cavity [4]; however, unlike humans, there is little evidence of pathogenicity.

Urinary tract infections (UTIs) are either temporary or permanent breaches in host defense mechanisms that allow microbes, mainly bacteria, to adhere, multiply, and persist within the urinary tract [5]. The main clinical features of UTI are dysuria, pollakiuria, and hematuria. These are most commonly caused by *Escherichia coli*; other uropathogens include Gram-positive cocci, *Proteus* spp. *Klebsiella* spp., *Pasteurella* spp., *Mycoplasma* spp., *Enterobacter* spp. and *Pseudomonas* spp. [6]. However, *P. acnes* has not been previously reported as a causative agent of UTI in dogs.

Here we report the presentation and clinical course of a UTI in a dog due to *P. acnes* infection.

## Case presentation

A 6-year-old female Japanese Shiba Inu was hospitalized in November 2014 for polyuria, polydipsia, severe hematuria, loss of appetite, weight loss, and lethargy. She had a recent history of hospital visits for cholestasis and hemorrhagic diarrhea due to severe whipworm infection. The owners granted permission for the publishing of this case report.

**Fig. 1:**
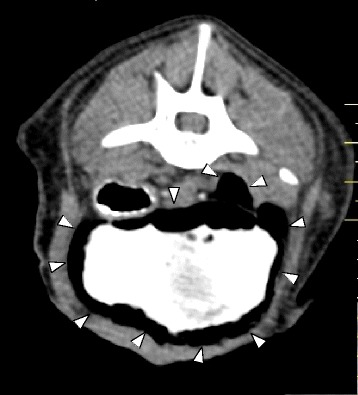
Computerized tomography of the bladder. Legend: Gas was visualized on the inner side of the bladder wall (*white arrowheads*). Urine contrast was enhanced by an imaging agent

At admission, blood tests revealed leukocytosis (3.79 × 10^9^/l with 90 % neutrophils), slight anemia (RBC 4.81 × 10^12^/l and PCV of 30.0 %), decreased albumin (1.5 g/dl), elevated hepatobiliary enzymes, possibly owing to cholestasis, and minimal changes in renal function (blood urea nitrogen 34.6 mg/dl and creatinine 0.5 mg/dl). No bacteria were detected in the peripheral blood via blood smear examination. Adrenocorticotropic hormone stimulation testing was negative. Thyroid hormone levels remained within the normal range. IgM antibody titers for coronavirus, adenovirus type 2, parvovirus, and canine distemper virus were below detectable limits (<3, 3, 256, and 512, respectively). Computerized tomography showed gas accumulation on the mucosal side of the bladder wall (Fig. 1).

**Fig. 2:**
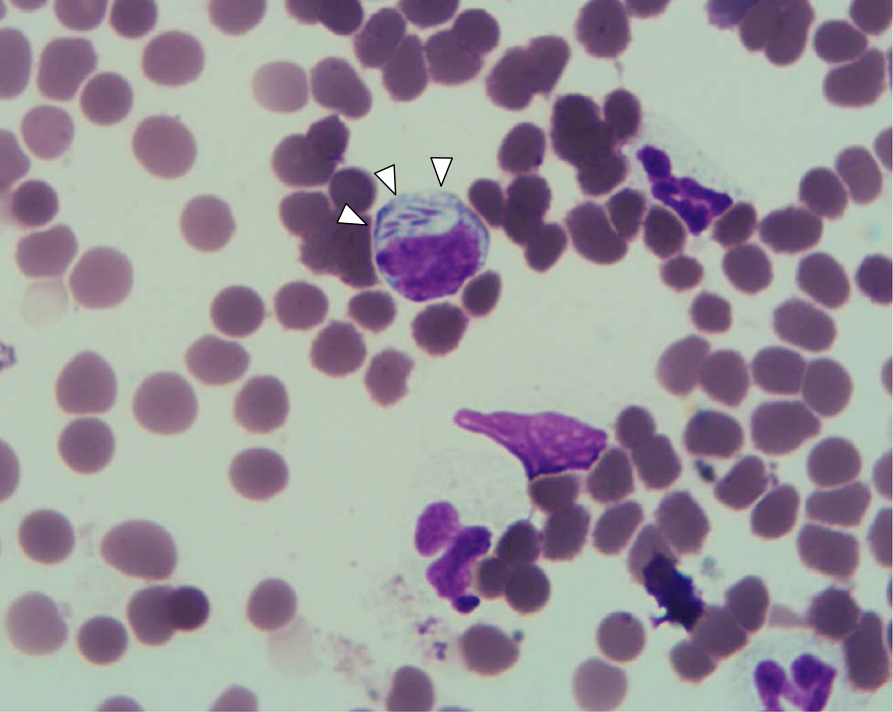
Urine sediment stained by Diff-Quik stain (× 1000). Legend: The rod-shaped bacterium was phagocytized by a monocyte (*white arrowhead*). Numerous erythrocytes were also observed

Urinalysis showed proteinuria and bilirubinuria without glycosuria. The urine sediment contained large numbers of erythrocytes and leucocytes including neutrophils and monocytes. Rod-shaped bacteria were also detected via Diff-Quik (Fig. 2) but not by Gram staining. No urinary calculi, yeasts or fungal hyphae were detected. Enrofloxacin (5 mg/kg of body weight once a day) and metronidazole (35 mg/kg twice a day) were administered empirically for the treatment of UTI.

On day 2, this case developed urinary retention and formed a blood clot in the bladder, which was found by echography. In addition, the patient’s renal function declined sharply (blood urea nitrogen 113.8 mg/dl and creatinine 3.0 mg/dl). After being informed of the dog’s condition, the owners decided to take the dog home; the patient died the next day. While a post-mortem examination would have been helpful in this case, this could not be performed as the owners declined post mortem examination.

Urine was obtained via catheterization on day 1 and plated on sheep blood agar (Eiken Chemical Co., Ltd., Tokyo, Japan) under aerobic and anaerobic conditions at 37 °C for 48 h. Small white colonies were observed only on plates incubated under anaerobic conditions; however, *Mycoplasma canis*, which can grow on blood agar plates [7], was not detected. The growing bacteria were non-spore-forming Gram-positive rods. The isolate was catalase-positive and identified as *P. acnes* using an API 20A (SYSMEX bioMérieux Co., Ltd., Tokyo, Japan) with a 99.9 % probability. Polymerase chain reaction (PCR) and DNA sequencing of the 16S rRNA gene confirmed the biochemical identification results. Multilocus sequence typing (MLST) using nine housekeeping genes (*cel*, *coa*, *fba*, *gms*, *lac*, *oxc*, *pak*, *recA*, and *zno*) was performed according to previously published protocols [8]. On the basis of these results, the isolate was determined to be of sequence type 53.

Susceptibility testing was performed by E-test (bioMérieux, Marcy l’Etoile, France) per the manufacturer’s directions against the following antimicrobials: ampicillin, amoxicillin/clavulanic acid, cefoxitin, imipenem, clindamycin, tetracycline, chloramphenicol, enrofloxacin, and metronidazole. Briefly, an inoculum was prepared by suspending a 48-h culture in reduced Brucella broth (Becton Dickinson Microbiology Systems, Cockeysville, MD, USA) to achieve a density of 1.0 on the McFarland nephelometer standard. The inoculum was plated on Brucella blood agar (bioMérieux Co., Ltd.). Minimum inhibitory concentrations (MICs) were determined following a 48-h incubation at 35 °C in an anaerobic chamber in accordance with Clinical and Laboratory Standards Institute guidelines [9]. *Bacteroides fragilis* ATCC 25285 was used as a reference strain. The bacterium showed high susceptibility to ampicillin, amoxicillin/clavulanic acid, cefoxitin, imipenem, clindamycin, tetracycline, chloramphenicol, and enrofloxacin, but was resistant to metronidazole (Table 1).

**Table 1:** Antimicrobial susceptibility of the *P. acnes* isolate

Antimicrobials	Minimum inhibitory concentration (μg/ml)
Ampicillin	0.125
Amoxicillin/clavulanic acid	0.063
Cefoxitin	0.25
Imipenem	0.031
Clindamycin	0.125
Tetracycline	0.125
Chloramphenicol	0.125
Metronidazole	>256
Enrofloxacin	0.5

## Discussion

There have been few reports of *Propionibacterium* infection in animals. Hodgin et al. [10] reported a case of a dog with osteomyelitis and arthritis due to *Propionibacterium* infection caused by a dog bite. In our case, there was no history of trauma and the route of infection could not be identified. Several factors that predispose to UTI, such as diabetes, Cushing’ disease, and hypothyroidism, were ruled out in this case based on urine and blood analyses. Anatomic abnormalities were not identified by diagnostic imaging. Concurrent infections with viruses, yeasts, fungi, and *M. canis*, were not identified by serological and microbiological tests. In addition, this patient did not have a history of steroid use. Thus, we could not identify any factors to predispose this patient to *P. acnes* UTI.

One hypothesis is that this was an ascending urinary tract infection, a common source of UTI [5], as *P. acnes* is as a part of the normal flora of the skin and feces. Another possibility is translocation of bacteria from the intestinal tract [3], given that the patient had concurrent severe diarrhea caused by whipworm infection, which could have damaged the gut/blood barrier. A final possibility is that the whipworm infection may have made the dog more susceptible to anthroponotic infection and the bacteria may have been normal flora from a human in contact with the dog. In this case, *P. acnes* was not detected in urine via Gram staining. This was also demonstrated in a previous study [11], and should be taken into account when considering *P. acnes* infection as a differential.

Our case was diagnosed as emphysematous cystitis (EC), a rare type of UTI, based on several diagnostic imaging techniques. EC occasionally occurs in diabetic dogs [12] but is relatively rare in nondiabetic dogs [13]. EC results from an infection by gas-producing bacteria, including *E. coli*, *Proteus* spp., *Aerobacter aerogenes*, and *Clostridium* spp*.* [14]. While *P. acnes* has not been reported to cause EC in dogs previously, gas production was noted in a human with *P. acnes* infection [15]. Thus, *P. acnes* should be regarded as a gas-producing bacterium and a potential cause for EC in dogs.

Our *P. acnes* isolate was identified as sequence type 53. This type has been isolated from human cases of acne and meningitis and reported in the MLST database [16]. In vitro antimicrobial susceptibility testing showed that our isolate was highly susceptible to most of the antimicrobials tested, except metronidazole, an agent to which *P. acnes* is consistently resistant [1]. A similar finding was reported for isolates from human cases of implant-associated infection [17]. Thus, our isolate shared sequence type and antimicrobial susceptibility with human isolates. However, the MLST database of *P. acnes* only contains human isolates, which prevents determination as to whether the bacteria were from a human source. The addition of *P. acnes* strains from dogs and other animals to the MLST database would be helpful in future epidemiological analyses.

This case’s condition rapidly worsened despite administration of enrofloxacin, to which the *P. acnes* isolate was susceptible. This case developed urinary retention due to the formation of blood clot, which is a risk factor for pyelonephritis [18]. In addition, in this case, renal function decreased concurrently with the development of urinary retention. Therefore, antimicrobial treatment may have had poor efficacy as a result of the development of acute pyelonephritis.

## Conclusion

UTIs are common bacterial infections in dogs. To the best of our knowledge, this is the first case report of a dog with UTI caused by *P. acnes*.

## Abbreviations

EC: emphysematous cyctitis
MIC: minimum inhibitory concentration
MLST: multilocus sequence typing
PCR: polymerase chain reaction
PCV: packed cell volume
RBC: red blood cell count
UTI: urinary tract infection
